# Differential Hydraulic Properties and Primary Metabolism in Fine Root of Avocado Trees Rootstocks

**DOI:** 10.3390/plants11081059

**Published:** 2022-04-13

**Authors:** Clemens P. Beyer, Cesar Barrientos-Sanhueza, Excequel Ponce, Romina Pedreschi, Italo F. Cuneo, Juan E. Alvaro

**Affiliations:** Facultad de Ciencias Agronómicas y de los Alimentos, Pontificia Universidad Católica de Valparaíso, Valparaíso 2340025, Chile; clemenspbeyer@gmail.com (C.P.B.); c.barrientos.sanhueza@gmail.com (C.B.-S.); excequel.p.g@gmail.com (E.P.); romina.pedreschi@pucv.cl (R.P.)

**Keywords:** *Persea americana* Mill. fine roots, root tip, primary metabolites, carbohydrates, water uptake, tree roots

## Abstract

Avocados (*Persea americana* Mill.) are one of the crops with the highest water footprints in Chile and the production is at risk due to severe and frequent droughts. The current production is mostly based on sexually (seed) propagated rootstocks, while clonally propagated rootstocks are on the rise. In a recent study, we found differences in aerial, root growth and water use efficiency between trees grown on these two different rootstocks under controlled continuous fertigation and environmental conditions. In this study, we further describe possible mechanisms which drive the differences. Avocado cv. “Hass” grafted on “Dusa” (D, clonally propagated) and “Mexicola” (M, sexually propagated) rootstocks and different root segments (3, 5 and 8 cm from root tip) were investigated using a combination of hydraulic measurements and polar metabolite (GC-MS) techniques. The results show significant differences in root hydraulic properties, indicating that “Mexicola” fine roots have higher water uptake capacity. The polar metabolites analysis revealed 13 compounds significantly different between rootstocks while nine were found significantly different among root segments. Principal component analysis (PCA) revealed differences between rootstocks and root segments. The data presented here highlight the importance of considering key physiological knowledge in avocado rootstocks breeding programs to be better prepared for future challenging environmental conditions.

## 1. Introduction

Future droughts will be more frequent, longer, and severe and it will be crucial to have more resilient agricultural systems [1]. Chile has been facing a mega-drought since 2010 that is depleting the water reservoirs in the central-northern part of the country [2]. Avocados (*Persea americana* Mill.) are one of the most important exporting fruits in Chile with an export volume of 120,000 tons in the season of 2019/2020 and a harvest area of 29,000 ha. At the same time, avocados are one of the crops with the highest water footprint (up to 1480 m^3^ t^−1^) produced in Chile [3]. As a result of the decrease of water availability, the production dropped by 40–50% in the season 2020/2021 [4].

In Chile, avocado orchards are mainly grafted onto sexually (seed) propagated avocado rootstocks (“Mexicola” and ‘Zutano’), while the clonal rootstocks are on the rise [5]. The most used clonal rootstocks (“Dusa”) are mostly chosen for their partial Phytophthora root rot resistance [6] but have a much higher production cost at nursery level. In previous studies [7,8], it has been shown that there are clear differences in the efficiency of those rootstocks in terms of water and nutrient uptake in comparison to root area, aerial growth, and main nonstructural carbohydrate accumulation. In one of these studies [7], the data suggested possible differential water uptake capacity between rootstocks. On the other hand, a recent study has shown good water stress response performance in “Dusa” rootstock [9].

Fine roots hydraulic properties have been extensively studied in the past [10]. Different root water uptakes in the tip and different root zones were found and modeled in lupine [11]. In citrus, different water uptakes in the root hierarchy were reported [12]. In grapevine rootstocks, clear differences have been reported in fine root structure and function during drought and recovery [13,14]. Techniques such as the root pressure probe can be a useful tool to compare different hydraulic properties [15]. Root traits are still insufficiently researched and are a promising resource for phenotyping and enhancing genetic material for future challenges such as climate change [16]. In this regard, a very powerful and common technique is the metabolomic analysis to understand plants response to stress, helping to better screen plants with a desired characteristic. Metabolomic analysis, for example, uses gas chromatography coupled to mass spectroscopy, with standards or reference libraries to quantify primary and secondary metabolites. Recent studies have incorporated its use in roots to account for differences between wheat and grapevine contrasting genotypes to drought resistance [17,18,19]. Therefore, the objective of this study was to combine root hydraulic and metabolomic tools to describe the differences between the roots and different root segments (tip and two proximal segments) of two avocado rootstocks (“Mexicola” and “Dusa”) in terms of root hydraulic properties, main nonstructural carbohydrates (fructose, glucose, sucrose, mannoheptulose, perseitol, starch) and other polar metabolites in a controlled environment.

## 2. Materials and Methods

### 2.1. Plant Material and Growth Condition

The avocado trees (cv. Hass) were three years and ten months old and were grafted on clonally propagated rootstock “Dusa” (D) and sexually propagated rootstock “Mexicola” (M). The trees were planted in a controlled environment at the facilities of the School of Agronomy of the Pontificia Universidad Católica de Valparaíso (PUCV) in the Province of Quillota (32°50′ S; 71°13′ O, 120 m.a.s.l), in the Region of Valparaíso, Chile. The controlled environment corresponded to a multi-tunnel greenhouse with trees planted in 55 L coir growth containers with drip irrigation. The avocado trees were continuously fertigated with a nutrient solution adjusted to the avocado plant. The electrical conductivity of the nutrient solution (exact composition in [7]) was set at 1.7 ± 0.1 dS·m^−1^ and the pH were held at 5.8 ± 0.1 [7]. The year average temperature and relative humidity inside the greenhouse corresponded to 17.4 °C and 71%, respectively.

### 2.2. Root Sampling and Root Hydraulic Measurements

The root development was observed in rhizotrons (windows of 20 × 45 cm with acrylic glass (4 mm thickness)) [7]. When there were sufficient roots, rhizotrons were opened and fine roots, ~2 mm diameter, of at least 8 cm length without laterals and with no presence of pathogens or evident damages were carefully extracted (eliminating substrate around the root with a paintbrush and cut with a scalpel), placed in water, and taken to the lab to perform the measurements. Then, each root was measured three times obtaining proximal successive cuts [20]. The first hydraulic conductivity (*Lp_r_*) measurement was performed with a root of ~8 cm. Then, ~2.5 cm of root was cut under water from the proximal end and *Lp_r_* was measured again. Finally, ~2.5 cm of root was cut under water from the proximal end again, leaving just the fine root tip, and *Lp_r_* was measured again. Three trees per treatment were sampled. A total of five roots per tree were measured. *Lp_r_* of excised fine roots was calculated using half-times (T_½_) of pressure exosmotic pressure-relaxation curves. The method consists of applying a pressure pulse with a syringe pump, increasing the root pressure equilibrium and maintaining the volume of the system constant until the equilibrium of the pressure is restored. It was calculated in the following way [15,21]:

k_wr_ = ln (2)/T_½_^w^ = Ar (ΔP_r_/ΔV_S_) *Lp_r_* (1)

where k_wr_ is the rate constant of water (and solutes) interchange through the root; T_½_^w^ is the half-time of the process; ΔP_r_/ΔV_S_ is the elastic coefficient of the measuring system (in MPa m^−3^), and A_r_ is the effective surface area of the root. The effective surface area (A_r_) was calculated using the diameter and the length of the root.

### 2.3. Analysis of Polar Metabolites and Main Nonstructural Carbohydrates

After the hydraulic conductivity measurements, the root segments were immediately frozen in liquid nitrogen and ground to a fine powder with an electric mill IKA-A11 (Staufen, Germany). Starch was digested overnight enzymatically following the protocol of [22] adapted by [23] adjusted to smaller samples. The generated soluble sugars were quantified with the DNS method. Results were expressed in mg·g^−1^ wet weight (*w*/*w*) starch. Main soluble sugars (fructose, glucose, sucrose, mannoheptulose and perseitol) and other polar metabolites were evaluated and quantified with gas chromatography coupled to mass spectrometry (GC-MS) with the methodology of [24] slightly modified by [25]. Main soluble sugars were expressed as mg·g^−1^ (*w*/*w*) and the other polar metabolites (mostly primary metabolism) of interest are expressed as relative abundance.

### 2.4. Data Analysis

Results of GC-MS included identification of the peaks by comparing retention time and mass spectra to a NIST14 library using Mass Hunter Quantitative software (Agilent Technologies, Santa Clara, CA, USA) and to an own home-built library. The relative response of each compound and the peak area data were corrected using the peak area of an internal standard, the sample weight, and a quality control (QC) (mixture of all samples). Found metabolites were analyzed by Principal Component Analysis (PCA) which was performed on auto scaled and centered data. Added where groups of the root segments and the rootstocks as data points and ellipses (95% confidence interval). Two-way ANOVA test and Tukey’s honest significant difference test (Tukey HSD) were used to determine significant differences among treatments and root segments combination. The statistical analysis and figures were made with R version 3.6.3 [26] using ggplot2 package [27].

## 3. Results

**Figure 1:**
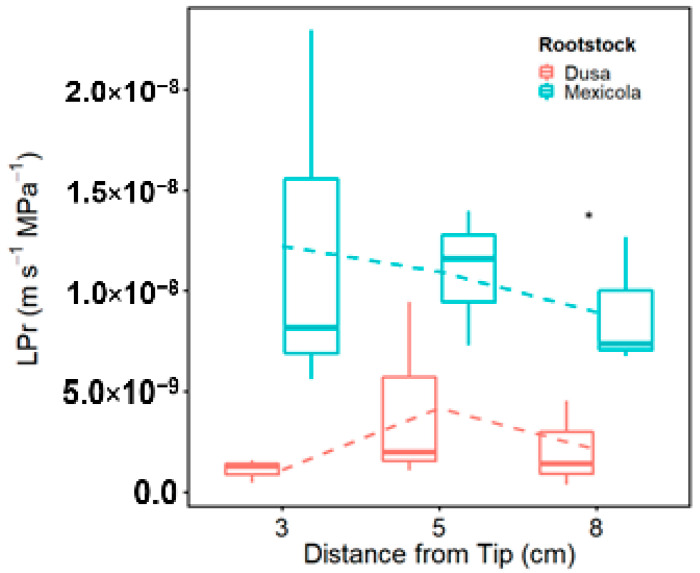
Fine root hydraulic conductivity (*LP_r_*) at different distances from the root tip (3, 5 and 8 cm) and in two rootstocks (“Mexicola” and “Dusa”). * Stands for significant differences (*p* > 0.05) of a two-way ANOVA test followed by a Tukey HSD.

In general, root hydraulic measurements displayed a higher hydraulic conductivity (*Lp_r_*) in fine roots of “Mexicola” (M) rootstock, showing significant differences between rootstocks in roots of 8 cm (Figure 1). In the case of M, the data showed a tendency of higher *Lp_r_* closer to the tip while, in the case of “Dusa” (D), the water uptake capacity was consistently low near the root tip (Figure 1).

**Figure 2:**
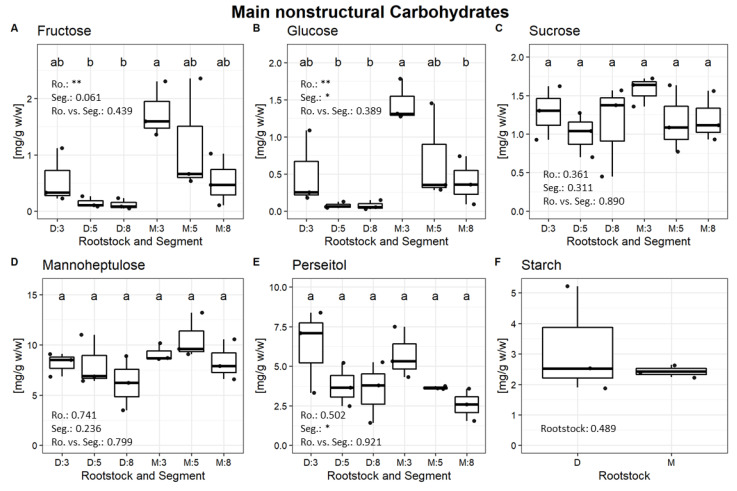
Main nonstructural carbohydrates (Fructose (**A**), Glucose (**B**), Sucrose (**C**), Mannoheptulose (**D**), Perseitol (**E**) and Starch (**F**)) plotted with a boxplot and results of a Tukey HSD test. Text are *p* values of the two-way ANOVA test (*p* < 0.05 (*), <0.01 (**)). “Dusa” (D) and “Mexicola” (M) rootstock and root segments (3, 5 and 8 cm). Due to sample limitation, for starch quantification, root segments were pooled.

Analysis of main nonstructural carbohydrates revealed significant differences in fructose and glucose levels between treatments and root segments (Figure 2).

**Figure 3:**
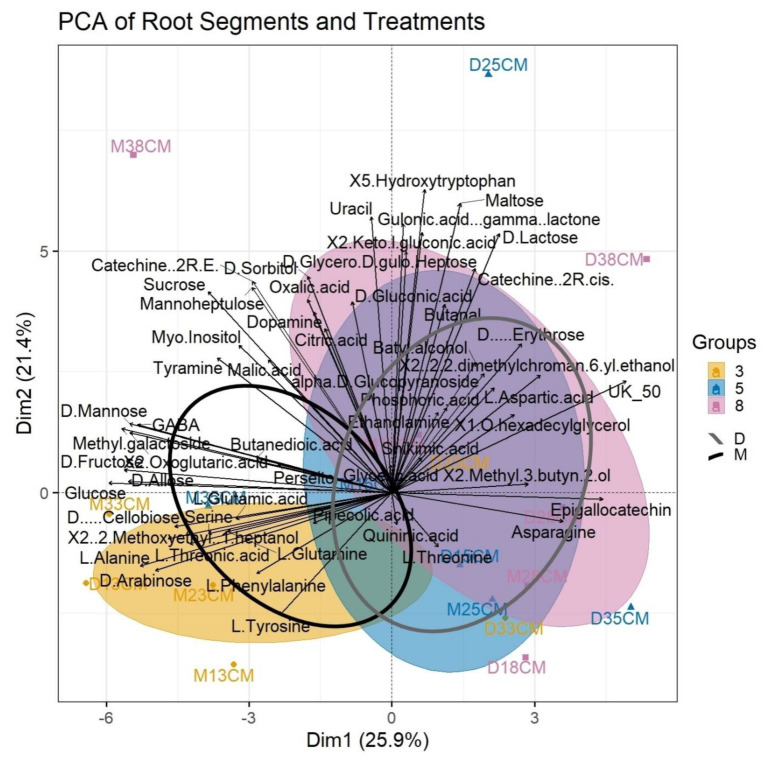
Principal component analysis (PCA) of polar metabolite analysis of the different root segments (3, 5 and 8 cm) and the two treatments (D, M). The shaded ellipses correspond to the root segments and the lined ellipses correspond to the treatments. The samples are separated by segment. The label is M or D (“Mexicola” or “Dusa”), 1, 2 or 3 (number of sample) and 3 CM, 5 CM or 8 CM (segment). The found compounds are: L–Alanine, Phosphoric acid, Butanedioic acid, Glyceric acid, Serine, Pipecolic acid, L–Threonine, Malic acid, L–Aspartic acid, GABA, L–Threonic acid, L–Glutamic acid, L–Phenylalanine, D–Arabinose, Asparagine, Shikimic acid, Citric acid, Quininic acid, D–Fructose, Tyramine, Glucose, L–Tyrosine, Dopamine, Myo–Inositol, D–Allose, 1–O–hexadecylglycerol, D–Glycero–D–gulo–Heptose, Sucrose, D–Lactose, Catechine (2R−cis), D–Sorbitol, Catechine (2R−E), Mannoheptulose, Perseitol, Oxalic acid, Ethanolamine, Uracil, 2–Methyl–3–butyn–2–ol, 2–Oxoglutaric acid, Unknown Compound 50 (UK_50), L–Glutamine, 2–Keto–l–gluconic acid, D–Gluconic acid, Methyl galactoside, Gulonic acid, .gamma.−lactone, 5–Hydroxytryptophan, 2–(2–Methoxyethyl)–1–heptanol, D–Mannose, D–(−)–Erythrose, Butanal, 2–(2,2–dimethylchroman–6–yl)ethanol, Batyl alcohol, alpha–D–Glucopyranoside, D–(+)–Cellobiose, Maltose and Epigallocatechin.

The concentration of those monosaccharides was higher in the root tip (3 cm) and in “Mexicola”, perseitol displayed a tendency of higher content while sucrose, mannoheptulose and starch showed no differences. GC-MS analysis of polar metabolites revealed 55 compounds mainly from primary metabolism and just a few from secondary metabolism (Figure 3). 

**Figure 4:**
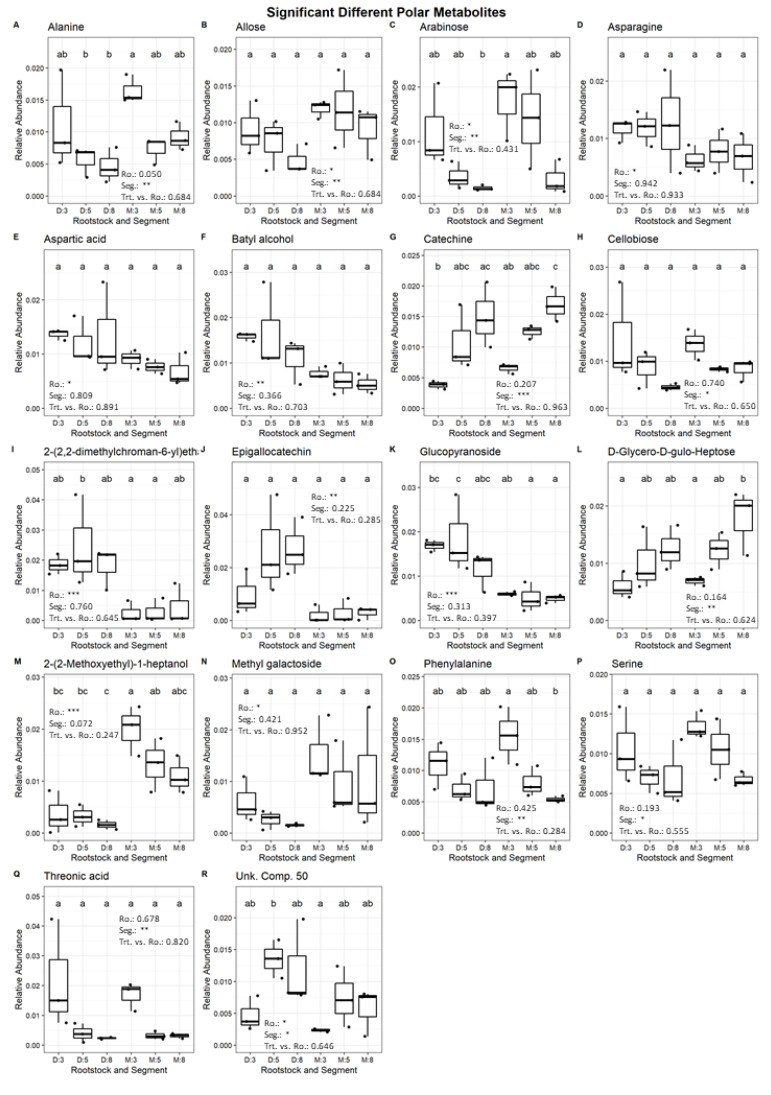
Boxplots of polar metabolites with significant differences (Alanine (**A**), Alose (**B**), Arabinose (**C**), Asparagine (**D**), Aspartic acid (**E**), Batyl alcohol (**F**), Catechin (**G**), Cellobiose (**H**), 2–(2,2–dimethylchroman–6–yl)ethanol (**I**), Epigallocatechin (**J**), Glucopyranoside (**K**), D–Glycero–D–gulo–Heptose (**L**), 2–(2–Methoxyethyl)–1–heptanol (**M**), Methyl galactoside (**N**), Phenylalanine (**O**), Serine (**P**), Threonic acid (**Q**) and Unknown Compound 50 (**R**)) between segments (3, 5 and 8 cm, Seg.) and/or treatments (“Dusa” or “Mexicola” Rootstock, Ro.) by a two-way ANOVA analysis. Text in plot are the p-values of the two-way ANOVA analysis (*p* < 0.05 (*), <0.01 (**), <0.001 (***)). Letters are results of Tukey’s HSD test.

Thirteen compounds (L-aspartic acid, D-arabinose, asparagine, D-fructose, D-glucose, D-allose, Unknown Compound-50, methyl galactoside, 2-(2-methoxyethyl)-1-heptanol, 2-(2,2-dimethylchroman-6-yl)ethanol, batyl alcohol, α-D-glucopyranoside, epigallocatechin) were significantly different between rootstocks and nine compounds (L-alanine, serine, L-threonic acid, L-phenylalanine, D-arabinose, D-glycero-D-gulo-heptose, catechin, Unknown Compound-50, D-cellobiose) were significantly different (Figure 4) among root segments. The PCA revealed two clusters (Figure 3). One of them contained mainly monosaccharides and some amino acids, and the other one is much larger and heterogenic (Figure 3). In the projection of treatment ellipses, it is possible to observe the association of M with the first cluster while D is more associated with the second cluster. When ellipses were separated by segments, differences between root tip (3 cm) and the other two root segments (5 and 8 cm) were found. The root tip associates with the first cluster of monosaccharides and some amino acids while the other root segments are better associated with the second cluster.

## 4. Discussion

The results presented here revealed significant differences in fine root water uptake capacity and metabolism between clonally propagated rootstock “Dusa” (D) and sexually propagated rootstock “Mexicola” (M). Differential water uptake capacity of single roots supports the findings of [7,8] where even though the water uptake was the same in both rootstocks, the root area of D was significantly higher. Our results highlight that the fine roots of M are more efficient absorbing water than the fine roots of D and suggest that a higher root area in D is probably related to a physical protection mechanism via callose deposition and lignification against soil pathogens rather than to resource acquisition [6,13,14,28,29]. Actual studies have shown that D has multiple adaption mechanisms to water stress [9] which may explain the higher resource consumption. The limited water uptake capacity of D is compensated by higher root growth area, compromising aerial development and carbohydrate accumulation [7,8].

The differences in water uptake capacity between both rootstocks might be connected to differential metabolism resulting in different suberin-lignin deposition in the endodermis and exodermis along the length of these rootstocks [13,14,28]. Our data suggest that fine roots of D are better sealed along the length and closed to the tip. The prediction and understanding of the water uptake for the root length, specific segment, hierarchy and structure is necessary to evaluate in more species, such as a study in Lupine that had shown a correlation between root length and water uptake [11]. These kinds of mechanisms can be understood as an adaptation against drought events such as suberin-lignin deposition, root biomass and architectural adaption [29]. Different genotypes can obtain different stress tolerance metabolism and different resource allocation [16]. Some metabolites (e.g., fructose, glucose, aspartic acid, inositol) coincide with our significant findings in avocado rootstocks in the present and former study [7]. In soybeans, a high sensitivity of the root tip to drought and flooding was found. There were especially effects on the carbohydrate metabolism [30]. In addition, in grapevine, it was found that there was an overall positive effect on amino acids, sugar and sugar alcohols under abiotic stress [31]. In our study, it was possible to see the importance of carbohydrate and amino acid metabolism in the root tip. M accumulates, as it does in previous studies [7], more carbohydrates (fructose, glucose, allose, arabinose). A special role could have the significant perseitol content in the root tip and its role in boron transport [32]. The importance of carbohydrates and amino acids was also shown in the root tips of beans associated in the lignification process and osmotic stress response [33]. Significantly higher phenylalanine (Figure 4) contents in the root tip as lignin and flavonoids precursor are of high interest through the structural abilities of lignin and its response to drought and the antimicrobial abilities of isoflavonoids which change under drought the expression in the root elongation zone [31,34,35]. In addition, in sweet cherry, significantly more asparagine was found in D as it has been reported to be a protective metabolite to osmotic stress and both asparagine and phenylalanine have been reported as potential biological markers for drought resistance [35]. Contrasting this is the significant higher content of epigallocatechin and glucopyranoside (Figure 4) in the proximal root parts of D, which could be a resource distribution in the biotic defense mechanism as a precursor for antifungal and antibacterial substances [36,37]. Perennial root systems are challenged to maintain the availability of metabolic energy while sustaining functionality and growth [31]. Further studies should explore the developmental anatomy along fine roots for both rootstocks in a quantitative and qualitative way under abiotic stress. Additionally, further analysis of metabolite profile should be completed to link it with differences in structure and function. There are still compounds with a significant unknown role (e.g., 2-(2-Methoxyethyl)-1-heptanol). The implementation of various omics approaches, combined as root omics, could be fruitful for future understanding of differential root properties [37]. This could be leading to a fundamental understanding of different mechanism adaptions in avocado rootstocks and other trees.

## 5. Conclusions

The present study investigated the physical and metabolomic differences between avocado rootstocks and different root sections. There found differences in the hydraulic conductivity between rootstocks and root parts. Furthermore, differences in the present metabolites were found between root sections and rootstocks. The results presented here highlight the need of including key physiological information (i.e., water uptake capacity) and metabolomic information in avocado rootstock breeding programs. In addition, our results suggest that more fine roots are not necessarily a synonym of more resource acquisition and might reflect a considerable difference in terms of efficiency of resource allocation. They could also be advantageous under biotic and abiotic stress conditions, depending on the agricultural contexts. These results could be adapted to other tree species and improve analysis of differences in physiological advantages between roots and rootstocks. The combination of the used techniques can drive innovation in the root omics area and open new opportunities for a deeper understanding of roots.

## Data Availability

All relevant data is shown in the figures. All raw data of chromatog.
